# Differential Involvement of ACKR3 C-Tail in β-Arrestin Recruitment, Trafficking and Internalization

**DOI:** 10.3390/cells10030618

**Published:** 2021-03-11

**Authors:** Aurélien Zarca, Claudia Perez, Jelle van den Bor, Jan Paul Bebelman, Joyce Heuninck, Rianna J. F. de Jonker, Thierry Durroux, Henry F. Vischer, Marco Siderius, Martine J. Smit

**Affiliations:** 1 Amsterdam Institute for Molecular and Life Sciences (AIMMS), Division of Medicinal Chemistry, Faculty of Science, Vrije Universiteit Amsterdam, De Boelelaan 1108, 1081 HZ Amsterdam, The Netherlands; aurelien.zarca@gmail.com (A.Z.); c.v.perezalmeria@vu.nl (C.P.); j.vanden.bor@vu.nl (J.v.d.B.); j.bebelman@vu.nl (J.P.B.); JONriaDE@hotmail.com (R.J.F.d.J.); h.f.vischer@vu.nl (H.F.V.); m.siderius@vu.nl (M.S.); 2 Institut de Génomique Fonctionnelle (IGF), Université de Montpellier, CNRS, INSERM, 34094 Montpellier, France; joyce_heuninck@hotmail.com (J.H.); Thierry.Durroux@igf.cnrs.fr (T.D.)

**Keywords:** GPCR, ACKR3, chemokine receptor, protein phosphorylation, β-arrestins, bioluminescence energy transfer (BRET), homogeneous time resolved fluorescence (HTRF), internalization, protein recruitment, GRKs

## Abstract

**Background**: The atypical chemokine receptor 3 (ACKR3) belongs to the superfamily of G protein-coupled receptors (GPCRs). Unlike classical GPCRs, this receptor does not activate G proteins in most cell types but recruits β-arrestins upon activation. ACKR3 plays an important role in cancer and vascular diseases. As recruitment of β-arrestins is triggered by phosphorylation of the C-terminal tail of GPCRs, we studied the role of different potential phosphorylation sites within the ACKR3 C-tail to further delineate the molecular mechanism of internalization and trafficking of this GPCR. **Methods**: We used various bioluminescence and fluorescence resonance energy transfer-based sensors and techniques in Human Embryonic Kidney (HEK) 293T cells expressing WT or phosphorylation site mutants of ACKR3 to measure CXCL12-induced recruitment of β-arrestins and G-protein-coupled receptor kinases (GRKs), receptor internalization and trafficking. **Results**: Upon CXCL12 stimulation, ACKR3 recruits both β-arrestin 1 and 2 with equivalent kinetic profiles. We identified interactions with GRK2, 3 and 5, with GRK2 and 3 being important for β-arrestin recruitment. Upon activation, ACKR3 internalizes and recycles back to the cell membrane. We demonstrate that β-arrestin recruitment to the receptor is mainly determined by a single cluster of phosphorylated residues on the C-tail of ACKR3, and that residue T^352^ and in part S^355^ are important residues for β-arrestin1 recruitment. Phosphorylation of the C-tail appears essential for ligand-induced internalization and important for differential β-arrestin recruitment. GRK2 and 3 play a key role in receptor internalization. Moreover, ACKR3 can still internalize when β-arrestin recruitment is impaired or in the absence of β-arrestins, using alternative internalization pathways. Our data indicate that distinct residues within the C-tail of ACKR3 differentially regulate CXCL12-induced β-arrestin recruitment, ACKR3 trafficking and internalization.

## 1. Introduction

Atypical chemokine receptors (ACKRs) are part of the superfamily of seven transmembrane receptors known as G-protein-coupled receptors (GPCRs). These receptors have been coined atypical, since, unlike classical GPCRs, they do not signal via G proteins upon activation [1,2]. ACKRs are believed to scavenge their ligands via internalization to modulate the chemokine availability for canonical chemokine receptors, making them chemotactic modulators, influencing many processes such as cell migration, immune response and inflammation [3]. Not surprisingly, ACKRs contribute to the progression of cardiovascular diseases and cancer [3,4,5,6,7].

The atypical chemokine receptor 3 (ACKR3, formerly known as CXCR7) is a GPCR sharing endogenous ligands with chemokine receptors CXCR3 (CXCL11) [8] and CXCR4 (CXCL12) [9]. This receptor has been demonstrated to be involved in, for example, brain development [10], cell migration [11], cancer [7] and cardiovascular diseases [12]. Yet, the molecular mechanisms by which ACKR3 contributes to these processes is unclear. Since it does not seem to signal directly via G proteins in most cell types, with the exception of astrocytes [13], the receptor acts as a β-arrestin biased GPCR [14]. For classical GPCRs, β-arrestins act as adaptor proteins, facilitating desensitization via internalization into clathrin-coated pits [15]. Some of these receptors display β-arrestin-independent internalization [16,17]. Beside their role as GPCR regulators, β-arrestins are also involved in downstream signaling, notably via the mitogen activated protein kinase (MAPK) pathway [18]. Since the atypical receptor ACKR3 does not activate G proteins in most cell types, β-arrestins are thought to play a major role in ACKR3 signaling and functionality [19,20]. Yet, recent studies are nuancing those results [10,21]. GPCR phosphorylation is a key regulatory mechanism of receptor activity [22]. Given that phosphorylation of the C-terminal tail (C-tail) of GPCRs by various kinases precedes β-arrestin recruitment to receptors [22,23], the molecular mechanisms by which C-terminal phosphorylation determines ACKR3 downstream events still needs further clarification [10,24,25]. Differential phosphorylation of residues of the receptor’s C-tail via different protein kinases, a process referred to as barcoding, can create different outcomes for the agonist-stimulated GPCR and determines the fate of the receptor, via selective protein recruitment, internalization and trafficking [26,27]. Some residues of the C-tail of ACKR3 appear as putative phosphorylation sites based on potential consensus sequences recognized by kinases [10], detected by mass-spectrometry [28,29,30,31] and some validated by phosphorylation studies and western blot analysis [10].

In this study, we show that upon CXCL12 stimulation, ACKR3 recruits both β-arrestin 1 and 2 with equivalent kinetic profiles, via G-protein-coupled receptor kinase (GRK) 2 and 3. ACKR3 internalizes and thereafter, recycles back to the cell surface. We demonstrate that β-arrestin recruitment to the receptor is potentially determined by a single phosphorylation-cluster on the C-tail, and that single residues serve as key phosphorylation sites for β-arrestin1 recruitment. Although phosphorylation of the C-tail appears essential for ligand-induced internalization and important for β-arrestin recruitment, agonist-induced internalization of ACKR3 can also occur in the absence of β-arrestins.

## 2. Materials and Methods

### 2.1. Materials

CXCL12 was obtained from Almac. CMPD101 was obtained from Tocris Bioscience. Hanks’ balanced salt solution, Dulbecco’s Modified Eagle medium and penicillin/streptomycin were obtained from Gibco. Fetal bovine serum was obtained from Bodinco (Alkmaar, Netherlands). Coelenterazine-h was obtained from Promega (Madison, WI, USA).

### 2.2. Cell Culture

Human Embryonic Kidney (HEK)293T cells (ATCC) and Clustered Regularly Interspaced Short Palindromic Repeats (CRISPR) HEK293 β-arrestin1/β-arrestin2 KO cells [32,33] were grown at 37 °C and 5% CO_2_ in Dulbecco’s Modified Eagle medium (Gibco, Waltham, MA, USA) supplemented with 10% fetal bovine serum (Bodinco), 50 IU/mL penicillin, and 50 mg/mL streptomycin (Gibco).

### 2.3. Generation of ACKR3 Mutants

N-terminus HA-tag and alanine substitution of all serine and threonine residues in the C-tail were introduced by DNA synthesis followed by PCR-based site-directed mutagenesis. The PCR fragments were cloned using internal BamHI and NotI restriction sites into a pcDEF_3_ plasmid already containing the RLuc8 gene with an in frame NotI restriction site at the 5′ end. SNAP-tag ACKR3 mutant constructs were generated from a pcDNA_3.1_ SNAP-tag ACKR3 plasmid kindly provided by Thierry Durroux (Montpellier, France) by cloning the promoter, the SNAP-tag and the N-terminus of ACKR3 into the pcDEF_3_ HA-tag ACKR3 construct using internal MluI and BstXI restriction sites. Following, the mutant C-tails were introduced using internal BstXI and NotI restriction sites from the HA-tag constructs.

### 2.4. Bioluminescence Resonance Energy Transfer (BRET)-Based Recruitment Assay

0.4 µg of pcDEF_3_-hACKR3-RLuc [19] or pcDEF_3_-hACKR3mut-RLuc and 1.6 µg of the completing BRET pair (pcDEF_3_-β-arrestin1-eYFP, pcDEF_3_-β-arrestin2-mVenus [34], pcDEF_3_-GRK2-mVenus, pcDEF_3_-GRK3-mVenus, pcDEF_3_-GRK5-mVenus, pcDEF_3_-GRK6-mVenus [35], Rab5a-mVenus, Rab7a-mVenus, Rab11-mVenus, K-Ras-mVenus [36,37]) plasmids were combined to 12 µg of PEI in a total volume of 250 µL 150 mM NaCl and incubated for 20 min at room temperature. One million resuspended HEK293T or CRISPR HEK293 β-arrestin1/β-arrestin2 KO cells were added to the DNA/PEI mix, and cells were subsequently seeded (30,000 cells per well) in a poly-L-lysine coated (Sigma, Saint-Louis, MO, USA) 96-well white plate (PS, F-bottom, white; Greiner Bio One, Kremsmünster, Austria). Two days after transfection, culture medium was substituted with Hanks’ balanced salt solution (Gibco). Next, cells were pre-incubated in Hanks’ balanced salt solution for 30 min, in presence or absence of 10 µM CMPD101 (Tocris, Bristol, UK) before addition of 2 µM Renilla Luciferase substrate coelenterazine-h (Promega). After 10 min, RLuc (480/20 nm) and BRET (540/40 nm) signals were measured on the Mithras LB940 (Berthold Technologies, Bad Wildbad, Germany) or Pherastar FS (BMG Labtech, Ortenberg, Germany) with the BRET1 module (BMG Labtech) and cells were stimulated with 100 nM CXCL12 (Almac, Craigavon, UK) before measuring in real-time. BRET ratios were calculated as: BRET/Rluc signal. Fold vehicle ratio was calculated as: BRET ratio (stimulated)/BRET ratio (vehicle). Data was analyzed using Prism 7 (GraphPad, San Diago, CA, USA).

### 2.5. Diffusion-Enhanced Resonance Energy Transfer (DERET)-Based Internalization Assay

HEK293T cells were transfected in the 96-well plate (Greiner Bio One, PS, F-bottom, white) using lipofectamine2000 (ThermoFisher, Waltham, MA, USA) agent according to manufacturer’s protocol. Ninety-six-well plates were coated with poly-ornithine (Sigma). For each well, a total of 200 ng of DNA (2 ng pcDNA_3.1_-SNAP-hACKR3-RLuc combined with 198 ng pcDEF_3_ plasmid) was diluted in optiMEM (ThermoFisher) to have a total volume of 25 µL. To this, 0.5 µL lipofectamine2000 mixed with 24.5 µL optiMEM was added. The transfection mixture was then incubated for 15 min at room temperature. Fifty µL of this mix was added to each well and 100 µL of medium containing 25,000 HEK293T cells was added on top of the transfection mixture. Two days after transfection, plates were washed twice with 100 µL TagLite^®^ buffer (Cisbio, Codolet, France) and incubated with 75 µL 100 nM SNAP-Lumi4-Tb (Cisbio) at 16 °C to block constitutive internalization for at least 1 h 30. Plates were then washed four times with 100 µL TagLite^®^. Ninety µL 25 µM fluorescein (Cisbio) (diluted in TagLite^®^) was added per well and 10 µL buffer or CXCL12 solution (ten times concentrated) were added per well. Internalization was followed as a ratio of 620/520 nm while exciting Lumi4-Tb at 340 nm and measured on the Infinite F500 plate reader (TECAN, Männedorf, Switzerland). 620/520 ratios were normalized to values between 0 and 1. Data was analyzed using Prism 7 (GraphPad).

### 2.6. Western Blot

Western blots were performed as previously published [38]. Briefly, cells were lysed with RIPA buffer (25 mM Tris-HCl (pH 7.4), 150 mM NaCl, 0.1% SDS, 1% (*v*/*v*) NP40, 0.5% Na-deoxycholate supplemented with 1 mM NaF, 1 mM NaVO4 and cOmplete protease inhibitor cocktail (Roche, Basel, Switzerland), sonicated and centrifuged to remove insoluble cell debris. Protein content of the lysates was determined using a bicinchoninic acid assay (BCA) protein estimation assay according to manufacturer’s instructions (Thermo Scientific, Waltham, MA, USA). Western blot was performed according to standard procedure and blotted against: β-arrestin1/2 (#4674 1:1000 in 5% milk powder; Cell Signaling, Danvers, MA, USA), β-actin (A5316 1:1000 in 5% BSA; Sigma-Aldrich, Saint-Louis, MO, USA). The western blot images were acquired by the use of a ChemiDoc imager (Bio-Rad, Hercules, CA, USA) and quantified by densitometry using Image Lab software (Bio-Rad).

## 3. Results

### 3.1. ACKR3 Recruitment of β-Arrestins

**Figure 1:**
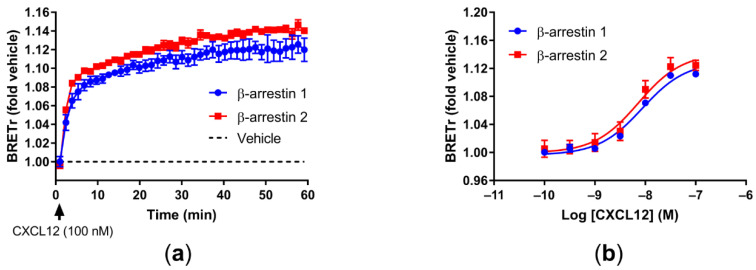
β-arrestin1 and β-arrestin2 recruitment to ACKR3 upon CXCL12 stimulation. (**a**) CXCL12-induced β-arrestin1 and β-arrestin2 recruitment to ACKR3 over time as measured by BRET in ACKR3 WT HEK293T cells; (**b**) Agonist-induced β-arrestin1 and β-arrestin2 recruitment to ACKR3 by increasing concentrations of CXCL12 as measured by BRET in HEK293T cells. Representative curves of three independent experiments with triplicates shown as mean ± SD.

To study CXCL12-induced β-arrestin recruitment by ACKR3, we expressed wild type ACKR3 fused with Renilla luciferase (RLuc) on the C-terminal tail (ACKR3-RLuc) as well as β-arrestin1 tagged with the eYFP fluorescent protein (βarrestin1-eYFP), or β-arrestin2 tagged with the mVenus fluorescent protein (βarrestin2-mVenus), creating a Bioluminescent Resonance Energy Transfer (BRET) pair sensor [39] in HEK293T (human embryonic kidney) cells. As previously described [40], ACKR3 recruited β-arrestin1 and β-arrestin2 upon CXCL12 stimulation (Figure 1a). The kinetics of recruitment of both β-arrestins to ACKR3 were similar, with a rapid association phase in the first ten minutes, followed by a slow-increasing plateau phase (Figure 1a). pEC_50_ values are also similar for the two isoforms (8.1 vs. 8.2) (Figure 1b, Table 1).

### 3.2. ACKR3 Recruitment of GRKs

**Figure 2:**
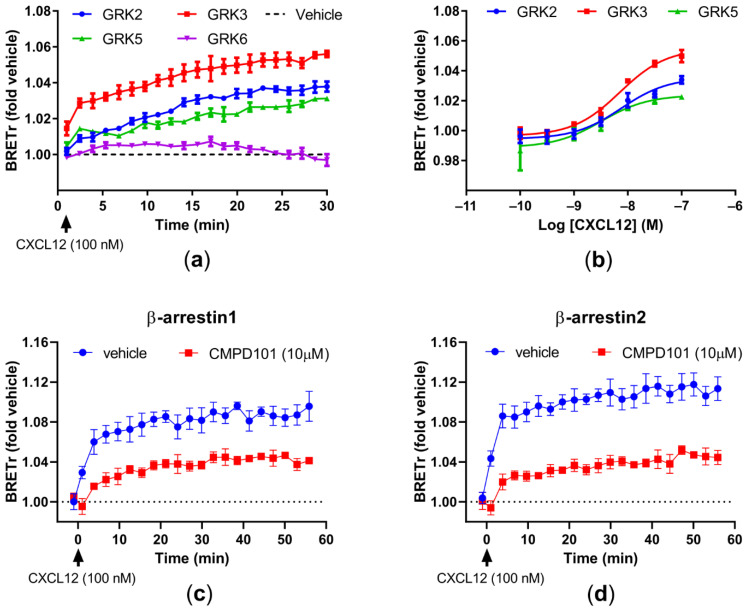
Influence of GRKs recruitment to ACKR3 upon CXCL12 stimulation. (**a**) CXCL12-induced GRKs recruitment to ACKR3 over time as measured by BRET in HEK293T cells; (**b**) Agonist-induced GRKs recruitment to ACKR3 by increasing concentrations of CXCL12 as measured by BRET in HEK293T cells after 30 min stimulation; (**c**) CXCL12-induced β-arrestin1 recruitment to ACKR3 over time, in presence or absence of 10 µM compound101 as measured by BRET in HEK293T cells; (**d**) CXCL12-induced β-arrestin2 recruitment to ACKR3 over time, in presence or absence of 10 µM compound101 as measured by BRET in HEK293T cells. Representative curves of three independent experiments with triplicates shown as mean ± SD.

Since recruitment of the β-arrestins to ACKR3 upon activation is triggered by a change in the phosphorylation pattern of the C-tail of the receptor [23], we investigated whether GRKs, proteins involved in GPCR phosphorylation, play a role in ACKR3 activation [22]. We developed BRET sensors to test this hypothesis by fusing the GRK isoforms (GRK2, GRK3, GRK5 and GRK6) to the mVenus fluorescent protein (GRK-mVenus) [35]. Upon addition of CXCL12, ACKR3 clearly recruited GRK2 and GRK3 (Figure 2a,b). Interestingly, ACKR3 also recruits GRK5, a plasma membrane-bound kinase [41]. On the other hand, ACKR3 does not affect trafficking of GRK6 (see [35] for a positive control of the GRK6 sensor), another plasma membrane-bound kinase. The pEC_50_ values for recruitment of each of the GRKs are in a similar range as the pEC_50_ values for recruitment of the β-arrestins (Table 1). To explore the involvement of GRKs in the functionality of ACKR3, we decided to test the effect of CMPD101, a known specific inhibitor of GRK2/3 [42]. Inhibition of those GRKs, led to a decrease of β-arrestin1/2 recruitment to the receptor (Figure 2c,d).

### 3.3. ACKR3 Internalization upon Activation

**Figure 3:**
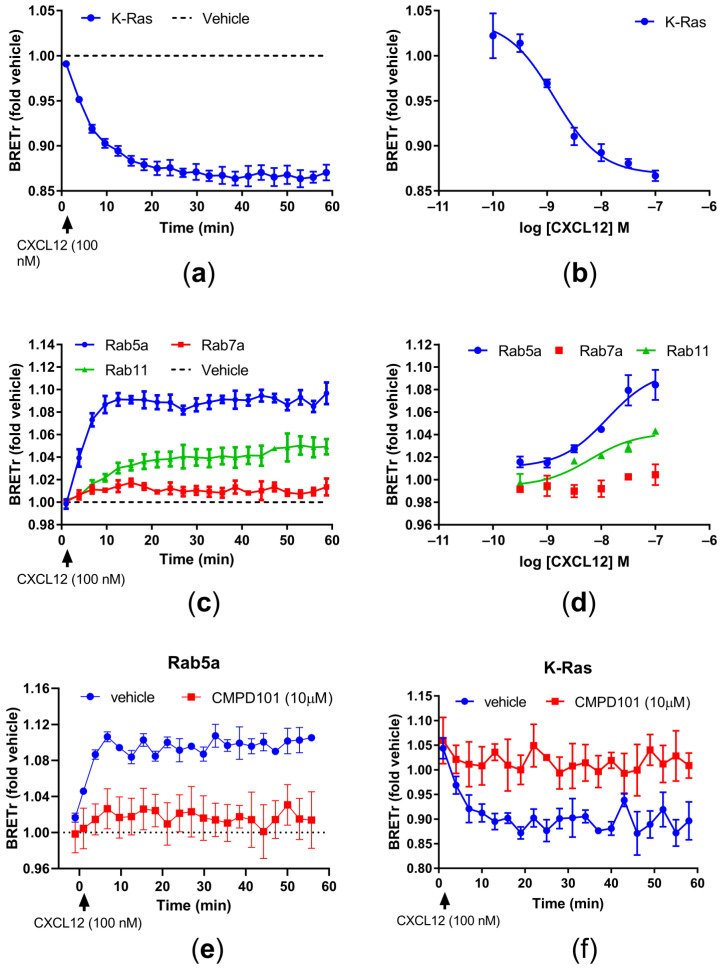
Internalization, recycling and/or degradation of ACKR3 upon CXCL12 stimulation. (**a**) CXCL12-induced K-Ras-Venus co-localization to ACKR3 over time as measured by BRET in HEK293T cells; (**b**) Agonist-induced K-Ras-Venus co-localization to ACKR3 by increasing concentrations of CXCL12 as measured by BRET in HEK293T cells after 30 min stimulation; (**c**) CXCL12-induced ACKR3 translocation to indicated Rab-Venus fusion proteins over time as measured by BRET in HEK293T cells; (**d**) CXCL12-induced ACKR3 translocation to indicated Rab-Venus by increasing concentrations of CXCL12 as measured by BRET in HEK293T cells after 30 min stimulation; (**e**) CXCL12-induced ACKR3 translocation to Rab5a-Venus over time, in presence or absence of 30 µM CMPD101 as measured by BRET in HEK293T cells; (**f**) CXCL12-induced K-Ras-Venus co-localization to ACKR3 over time, in presence or absence of 10 µM CMPD101 as measured by BRET in HEK293T cells. Representative curves of three independent experiments with triplicates shown as mean ± SD.

Since ACKR3 is considered a decoy or scavenging receptor [14], it is important to monitor trafficking of ACKR3 upon activation by CXCL12. To follow the receptor’s fate, we used BRET-based localization sensors [36,37] that are specific for different cell compartments, namely K-Ras (cell membrane) [43], Rab5a (early endosome) [44], Rab7a (late endosome) [45] and Rab11 (recycling endosome) [46]. ACKR3 is known to display constitutive internalization [19,20,24]. In this study, we focused on CXCL12-activated internalization of ACKR3 using these cell compartment specific BRET sensors. Upon CXCL12 activation (Figure 3), ACKR3 rapidly disappears from the cell membrane (K-Ras), and relocalizes to early endosome vesicles (Rab5a) (Figure 3a,c). CXCL12 does not seem to trigger degradation of the receptor since it does not co-localize with Rab7a (Figure 3c), a marker of late endosomes leading to a degradation pathway for proteins [47]. On the other hand, ACKR3 co-localizes with Rab11 in recycling endosomes, indicating recycling to the cell membrane. Interestingly, pEC_50_ values linked with internalization (Figure 3b,d) are one log unit higher than that of β-arrestin or GRK recruitment (Figure 1 and Figure 2; Table 1). Pre-treatment of the cells with CMPD101 in the Rab5a and K-Ras BRET assays (Figure 3e,f) showed that the receptor does not translocate to the early endosomes (Figure 3e), and remains at the plasma membrane (Figure 3f), implying impaired internalization. This points to a key involvement of GRK2 and/or GRK3 in the internalization process of ACKR3.

### 3.4. Involvement C-Tail Phosphorylation Sites ACKR3 in Differential Recruitment of β-Arrestins

**Scheme 1:**
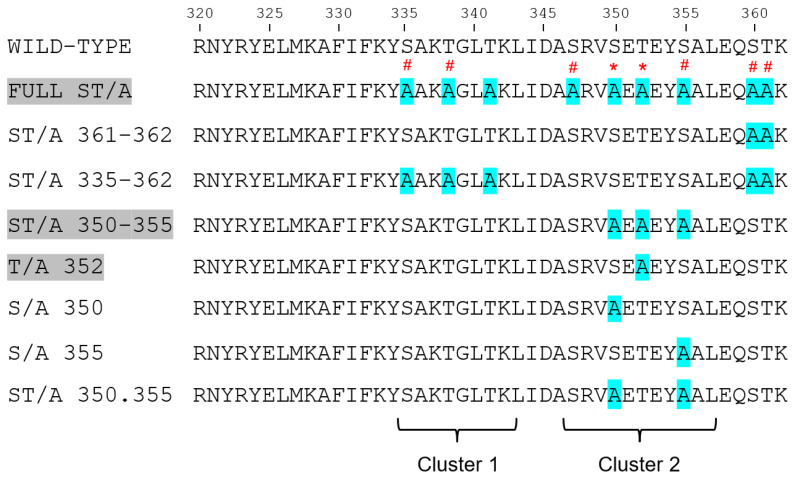
ACKR3 C-terminal alanine mutants. * Detected and validated phosphorylated residues [10]. # Detected phosphorylated residues by mass-spectrometry [28,29,30,31]. Highlighted mutants were also tested in internalization assays.

**Figure 4:**
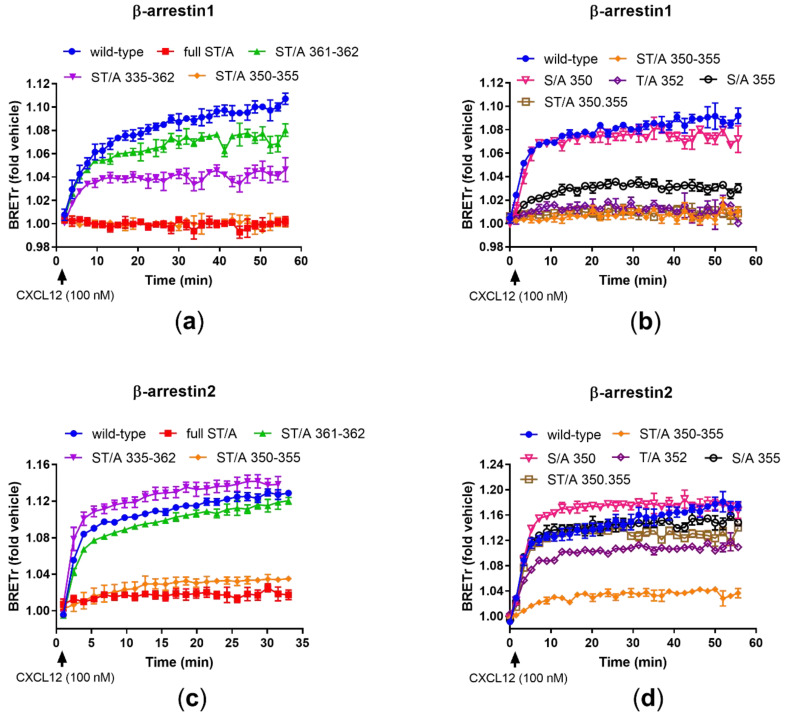
β-arrestin1 and β-arrestin2 recruitment to ACKR3 wild-type and mutant receptors upon CXCL12 stimulation. (**a**) CXCL12-induced β-arrestin1 recruitment to ACKR3 wild-type and different cluster mutant receptors over time as measured by BRET in HEK293T cells; (**b**) CXCL12-induced β-arrestin1 recruitment to ACKR3 wild-type and cluster 2 mutant receptors over time as measured by BRET in HEK293T cells; (**c**) CXCL12-induced β-arrestin2 recruitment to ACKR3 wild-type and different cluster mutant receptors over time as measured by BRET in HEK293T cells; (**d**) CXCL12-induced β-arrestin2 recruitment to ACKR3 wild-type and cluster 2 mutant receptors over time as measured by BRET in HEK293T cells. Representative curves of three independent experiments with triplicates shown as mean ± SD.

To investigate the role of phosphorylation of the C-terminus (C-tail) of ACKR3, we generated a set of mutant receptors by mutating potential phosphorylation sites, Serine (S) and Threonine (T) residues, to an Alanine (A) residue. We evaluated these mutants in the BRET-based β-arrestin recruitment assay. A decrease in BRET signal may indicate a decrease in protein recruitment and/or a decrease in BRET efficiency (donor energy transfer to acceptor) [48], due to changes in orientation of BRET pairs by the alanine substitution. Mutants showing marked effects on β-arrestin 1 and/or 2 BRET signal were investigated further (Scheme 1). The C-tail consists of two separate clusters of phosphorylation sites and two distal (S360/T361) residues (Scheme 1). When mutating all potential phosphorylation sites of the C-tail (full ST/A), ACKR3 neither recruits β-arrestin1 nor β-arrestin2 (Figure 4a–c), suggesting the prerequisite of phosphorylation for CXCL12-mediated β-arrestin recruitment. Mutating only the two distal residues (ST/A 361-362) of the C-tail did not result in any impairment of β-arrestin recruitment (Scheme 1, Figure 4a,c). Similarly, combining mutation of cluster 1 and the S360/T361 (ST/A 335-362) did not affect β-arrestin2 recruitment, but partially impaired the β-arrestin1 BRET signal (Figure 4a,c). Cluster 2 contains a GRK2 consensus phosphorylation site [10]. Mutating only S/T residues within phosphorylation cluster 2 (S350/T352/S355) in the so-called ST/A 350-355 mutant resulted, like the full ST/A mutant, in a total loss of β-arrestin recruitment (Figure 4a,c). Finally, by mutating only one residue (T352) within cluster 2 to an alanine (T/A 352), ACKR3 did not recruit β-arrestin1 at all, as seen for the full ST/A and ST/A 350-355 mutant (Figure 4a), contrary to S/A 350 (Scheme 1, Figure 4b,d).

The S/A 355 mutant showed impaired β-arrestin1 BRET signal. Yet, the T/A 352 and S/A 355 mutant were still able to recruit β-arrestin2 upon binding of CXCL12, with only slightly reduced BRET signal compared to WT ACKR3 for T/A 352 (Figure 4d). The initial kinetic phase of β-arrestin2 recruitment is similar, but T/A 352 did not show a continuously increasing plateau phase. These data indicate the differential role of phosphorylation sites in recruitment of β-arrestin1 and 2. Mutants T/A 352, ST/A 350-355 and full ST/A were deemed most interesting given their varied profiles in β-arrestin recruitment and therefore characterized further.

### 3.5. Involvement C-Tail Phosphorylation Sites ACKR3 in Internalization

**Figure 5:**
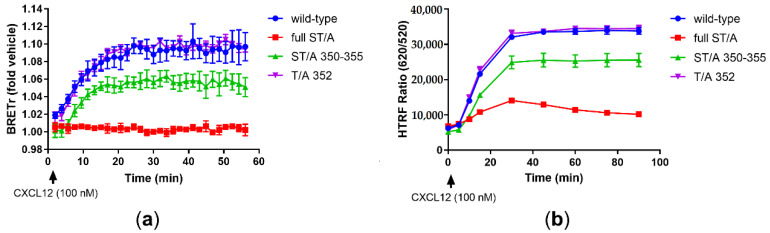
Agonist-induced internalization of ACKR3 wild-type and mutant receptors. (**a**) CXCL12-induced ACKR3 wild-type and mutant receptors translocation to Rab5a-Venus ACKR3 over time as measured by BRET in HEK293T cells; (**b**) CXCL12-mediated internalization of ACKR3 wild-type and mutant receptors over time as measured by TR-FRET in HEK293T cells. Representative curves of at least three independent experiments with triplicates shown as mean ± SD.

Since phosphorylation of the C-tail of ACKR3 seems to be playing an important role in β-arrestin recruitment, we studied its effect on CXCL12-mediated ACKR3 internalization. To monitor the internalization of the WT and mutant receptors, we used the BRET-based Rab5a sensor (Figure 3 and Figure 5a), and a TR-FRET (Time-Resolved Fluorescence Resonance Energy Transfer)-based DERET (Diffusion Enhanced Resonance Energy Transfer) approach (Figure 5b) to validate results in an orthogonal assay [49].

To enable the use of this technology with ACKR3, we fused a SNAP tag onto the N-terminus of WT and mutant receptors, to allow labeling by a fluorophore (Lumi4-Tb) and transfected HEK293T with these constructs. Labeling was performed at 16 °C to prevent protein trafficking in the cells ensuring that Lumi4-Tb will only label receptors initially present at the cell membrane, allowing detection of internalization events when exposing the cells to 37 °C, by a decrease of TR-FRET between the Lumi4-Tb and the non-cell penetrating fluorophore in solution. Upon stimulation by CXCL12 (Figure 5), the internalization kinetics of the WT, the T/A 352 and the ST/A 350–355 mutants are similar, albeit with a decrease in internalization rate when comparing ST/A 350/355 with WT. CXCL12 stimulation of the full ST/A mutant only resulted in marginal receptor internalization when using DERET technology (Figure 5b), and no internalization detected using the BRET sensor (Figure 5a). The full ST/A, ST/A 350–355 and T/A 352 ACKR3 mutants showed comparable expression levels in different experimental set ups (Figure S1) not explaining the observed differences. Thus, demonstrating that CXCL12-triggered phosphorylation of the C-tail is necessary for proper internalization of ACKR3, as shown by the removal of all phosphorylation sites.

### 3.6. Involvement β-Arrestins in ACKR3 Internalization

**Figure 6:**
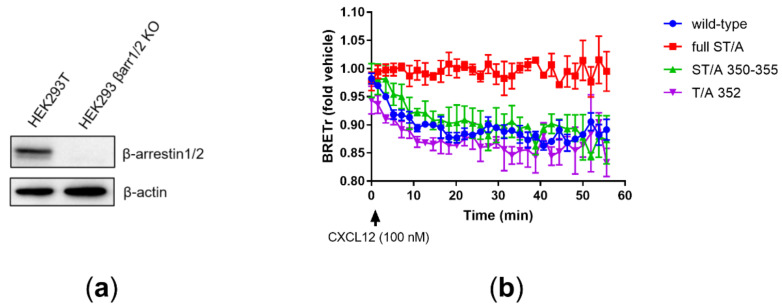
Internalization of ACKR3 wild-type and mutant receptors in a β-arrestin1/β-arrestin2 KO cell line upon CXCL12 stimulation. (**a**) Western Blot detection of β-arrestin1/β-arrestin2 expression in HEK293T cells and CRISPR HEK293 β-arrestin1/β-arrestin2 KO cells; (**b**) CXCL12-induced K-Ras co-localization with ACKR3 wild-type and mutant receptors over time as measured by BRET in CRISPR HEK293 β-arrestin1/β-arrestin2 KO cells. Representative curves of three independent experiments with triplicates shown as mean ± SD.

Since mutants defective in β*-*arrestin1/2 recruitment still showed internalization, we hypothesized that the internalization process of ACKR3 is not fully dependent on β*-*arrestin (Scheme 1, Figure 4a,b and Figure 5), we tested this hypothesis using HEK293T cells in which both β-arrestin1 and β-arrestin2 were knocked-out (KO) using CRISPR technology [32,33]. Transfecting the K-Ras BRET sensor, a cell membrane-bound protein, along with ACKR3 and the different mutants, we monitored internalization over time of the different receptors. Upon stimulation by CXCL12 (Figure 6), and as previously suggested [10,21], ACKR3 WT does internalize in the absence of β*-*arrestins, with similar kinetics as in WT HEK293T (Figure 3). As can be seen in Figure 6, the T/A 352 and ST/A 350–355 mutants are both internalizing in a similar manner as the WT receptor upon activation in the β-arrestin1/2 CRISPR KO cells. As anticipated from the previous internalization experiments (Figure 5), the lack of phosphorylation sites of the full ST/A mutant prevents it from internalizing when stimulated.

## 4. Discussion

Accumulating evidence indicates the involvement of ACKR3 in pathological processes, specifically in certain types of cancers and cardiovascular diseases [3,12]. ACKR3, along with CXCR4, with which it shares a ligand, CXCL12 [9], plays an important role in cancer progression [7,50]. In this study, we show that, upon CXCL12 stimulation, ACKR3 recruits GRK2/3/5 proteins and β-arrestin1 and 2 with similar kinetics, and internalizes rapidly in endosomal compartments following by its recycling to the membrane. GRK2/3 involvement in ACKR3 phosphorylation is further substantiated by inhibition of CXCL12 mediated β-arrestin1 and 2 recruitment in the presence of GRK2/3 inhibitor CMPD101. While investigating the impact of phosphorylation of the C-tail on ACKR3 function, we found that phosphorylation is important for recruitment of β-arrestins, internalization and receptor trafficking. Yet, the β-arrestins appear dispensable for internalization of ACKR3. The ^350^SETEYS^355^ cluster appears crucial for β-arrestin1 and 2 recruitment, with T^352^ and in part S^355^ serving as key residues for β-arrestin1 interaction.

Regulation of the ACKR3 lifecycle and signaling remains unclear. In this study, we demonstrate the importance of phosphorylation within the C-tail of the receptor influencing its behavior. The role of β-arrestins in ACKR3 function is still under debate, with distinct results using different cell types and techniques [10,19,20,51]. Here we show equivalent recruitment profiles of both β-arrestins in HEK293T cells upon overexpression. The sustained β-arrestin recruitment to the receptor upon CXCL12 activation could be exemplary for the role of ACKR3 sequestering β-arrestins from CXCR4 [52], thereby modulating indirectly CXCR4 activity.

We show that ACKR3 interacts with GRK2, 3 and 5 upon CXCL12 activation. This is partly in line with the results obtained by Stumm and colleagues [10], demonstrating that GRK2 plays a role in the phosphorylation of the C-tail of ACKR3. Considering that they only investigated the ^350^SETE^353^ cluster of the C-tail, which is a putative GRK2 phosphorylation consensus site [10], GRK3 and 5 could very well be involved in the phosphorylation of other residues within the C-tail of ACKR3. Interestingly, GRK5 is a plasma membrane-bound kinase [41]. Given that ACKR3 internalizes upon activation (Figure 3), a negative effect on the BRET ratio was expected to appear within a 20 min timeframe with membrane-bound GRK5 separating from the internalizing receptor. Yet, an increase of GRK5 recruitment is observed upon stimulation of ACKR3. Enhanced co-internalization of both ACKR3 and GRK5 in early endosomes could be an explanation for these observations. As recently reported, CMPD101 hampers β-arrestin2 recruitment via inhibiting GRK2 and 3 activity [10,53]. Here, we confirm their data, and furthermore demonstrate that those GRKs are also involved in the recruitment mechanism of β-arrestin1 and β-arrestin2. To finetune insight into phospho-barcoding within the ACKR3 C-terminus, involvement of other common GPCR kinases (PKA, PKC) and kinases identified in pulldown experiments of ACKR3 should be investigated as well [13].

As previously published, ACKR3 [10,19,20,24] constitutively internalizes, and we demonstrated it internalizes further upon CXCL12 activation (Figure 3 and Figure 5). BRET experiments of ACKR3 with Rab7a and Rab11 did not reveal agonist-mediated degradation of the receptor but indicated recycling to the plasma membrane. Investigating the role of the C-tail of the receptor and mutating its potentially phosphorylated residues, we generated clear evidence regarding the functional internalization process of the receptor which still occurs in the absence of β-arrestins. All residues of the ^350^SETEYS^355^ have been detected as being phosphorylated using mass-spectrometry analysis [29], with S^350^ and T^352^ phosphorylation being validated by western blot [10]. This is in contrast with earlier published data [19,20], but in line with recent experimental work [10,21], showing functional ACKR3 in the absence of β-arrestins. The differences in cellular background and between full (CRISPR KO approach) or partial inhibition of expression (siRNA approach) may in part explain the observed differences [54]. In this study, we see that agonist-mediated internalization is β-arrestin1 (T/A 352) and β-arrestin2 independent (ST/A 350–355). It is possible that β-arrestins are important for internalization of ACKR3, but in the absence or impairment of β-arrestin1 and 2, alternative internalization pathways may occur resulting in an internalizing receptor. Other GPCRs [16], such as M_2_ muscarinic receptor, angiotensin AT_1A_ receptor or formyl peptide receptor can also internalize in a β-arrestin independent manner. Instead, receptor internalization still occur via clathrin-coated pits [19,20,24], but via other adaptor proteins such as GRK2 [55] or AP-2 [56]. In our study, inhibition of GRK2 and 3 via CMPD101 (Figure 3e,f) showed a marked decrease in internalization of the receptor using Rab5a and K-Ras BRET assays. These data indicate that GRK2, known as an important signaling hub [57], and/or GRK3, are involved in ACKR3 internalization either via their kinase activity or scaffolding role. It will be of interest to further determine the roles of GRK2 and 3, and other kinases or proteins involved in ACKR3 internalization.

Another interesting finding of this study is that β-arrestin1 recruitment relies on the potential phosphorylation of T^352^ and S^355^ residues. Hence, these residues can serve as key phosphorylation sites that direct β-arrestin1 interaction and may serve as anchor points to switch on/off the recruitment of β-arrestin1. Recently, a single phosphorylation residue within the Vasopressin 2 receptor was found to serve as an anchor point for interaction with β-arrestin1 and 2 [58]. This key phosphorylation site was suggested to govern the stability of interaction with the β-arrestins regulating the interdomain rotation in β-arrestins [58]. Another plausible explanation could be a hierarchical phosphorylation of ACKR3 C-tail, like CXCR4 [59], where phosphorylation of T^352^ and S^355^ would be indispensable for the phosphorylation of further residues directly responsible for β-arrestin1 interactions. Here we have identified T^352^ as a crucial residue for β-arrestin1 recruitment, with the S^355^ residue having a lesser effect (Figure 4) and cluster 1 an even smaller one (Figure 4), and T^352^ only marginally affecting β-arrestin2 recruitment. The mutants T/A 352 and S/A 355 represent β-arrestin1 deficient mutants that can be used in the future to delineate the contribution of this isoform downstream of ACKR3. Since β-arrestins are not necessarily involved in internalization of the receptor, studying the impact of differential β-arrestin recruitment on functional consequences for downstream signaling of ACKR3 is of interest as β-arrestin functions do not always overlap [60,61], in particular regarding potential MAPK pathway activation [18]. Such studies could be complemented with silencing or knocking-down either β-arrestin isoform. It is known that β-arrestins bind in different ways to the receptor, either binding the C-tail, or engaging the core of the GPCR, which can lead to distinct functionality of the GPCR-βarrestin complex [62,63].

Phosphorylation of residues within the ^350^SETEYS^355^ (cluster 2) appears crucial for both β-arrestin1 and 2 recruitment, and much less for ^335^SAKTGLT^341^ (cluster 1) despite having some effect on β-arrestin1 interactions. There are clear differences of the contribution of phosphorylation sites within cluster 2, which could lead to differential recruitment of one β-arrestin isoform over another. It should be noted, however, that there is no evidence thus far of region-specific phosphorylation in different cell types upon agonist exposure. The ST/A 350–355 mutant serves as an ACKR3 β-arrestin1 and 2-deficient mutant, defective in β-arrestin1 and 2 recruitment, which may be of interest to dissect the importance of β-arrestin recruitment for ACKR3 function. Recently, the structure of β-arrestin2 in complex with the phosphopeptide of the ACKR3 C-tail ^335^SAKTGLT^341^ (cluster 1) has been resolved [64]. Binding of β-arrestin2 was directed by the PxxPxxP motif. It could be imagined that this cluster is responsible for the binding of the arrestins, whether it is phosphorylated or not, while, in accordance with our results, the ^350^SETEYS^355^ cluster is responsible for the differential regulation of the β-arrestins, and the C-tail in its entirety regulating internalization of the receptor. It is also noted that β-arrestin2 has been shown to be more flexible than β-arrestin1 [65,66], which may explain why mutation of some of the putative phosphorylation sites have a more pronounced effect on β-arrestin1 recruitment.

**Scheme 2:**
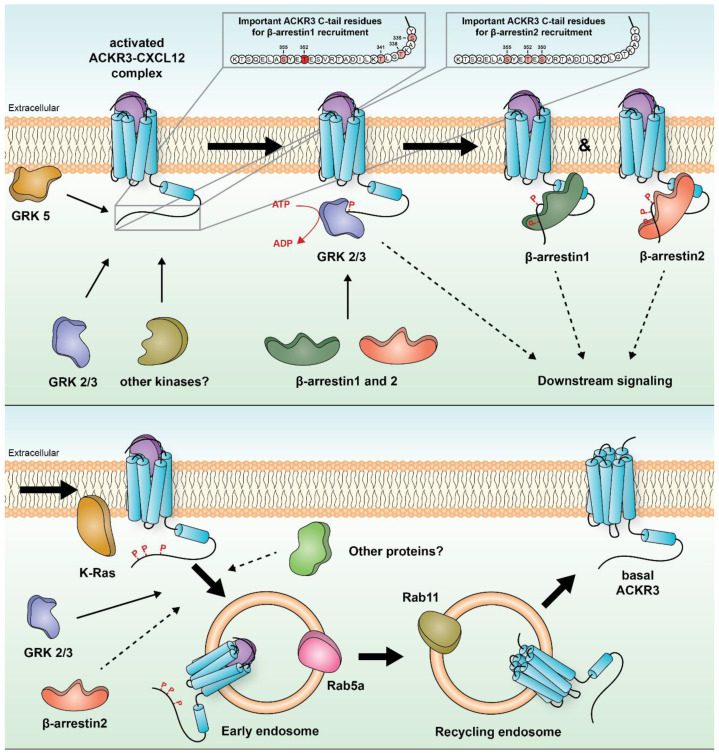
Proposed model for CXCL12-induced ACKR3 protein recruitment, trafficking and internalization. Upper panel: Upon stimulation via CXCL12, GRK2/3/5, along with potential other kinases, are recruited and phosphorylate the C-tail of the receptor. The phosphorylation of specific residues of the receptor’s C-tail is then recognized by β-arrestin1 and β-arrestin2, and/or other proteins to trigger downstream signaling. Lower panel: Activation of ACKR3 triggers internalization to the early endosome (Rab5a), via GRK2/3 and β-arrestin2, and other proteins independently from the canonical β-arrestins. It is then followed by recycling (Rab11) of the receptor to the cell surface.

In summary, we provide evidence of β-arrestin1/2 and GRK2/3/5 interactions with ACKR3 upon activation by its cognate ligand CXCL12 (Scheme 2). C-tail phosphorylation of the receptor is a key mechanism for regulation of ACKR3, selectively directing β-arrestin1 and 2 interactions, and internalization. Agonist-induced internalization of this receptor is GRK2- and/or 3-dependent, yet, in the absence of β-arrestins, ACKR3 still internalizes. Phosphorylation of the distinct sites within the C-tail differentially affects CXCL12-induced β-arrestin recruitment, and ACKR3 trafficking and internalization. Future studies should further delineate the functional consequences of ACKR3 interacting proteins on its function.

## Figures and Tables

**Table 1:** pEC_50_ of CXCL12 activating ACKR3 in various BRET assays.

BRET Results pEC_50_ ± SEM (*n* = 3)									
GRK2	GRK3	GRK5	GRK6	β-Arrestin1	β-Arrestin2	K-Ras	Rab5a	Rab7a	Rab11
8.2 ± 0.1	8.1 ± 0.0	8.3 ± 0.2	ND *	8.1 ± 0.1	8.2 ± 0.0	8.9 ± 0.1	9.1 ± 0.1	ND *	9.1 ± 0.1

All results are average ± SEM of three independent experiments. * Not detected.

## Data Availability

The data presented in this study are available in the article and the Supplementary Material.

## Supplementary Materials

The following are available online at https://www.mdpi.com/2073-4409/10/3/618/s1. Figure S1. Expression levels of ACKR3 wild-type and mutant receptors in various assays.
