# Supplementary material for: Differential Involvement of ACKR3 C-Tail in β-Arrestin Recruitment, Trafficking and Internalization
Source: Cells. 2021 Mar 11;10(3):618. doi: 10.3390/cells10030618 (PMC8002179; doi:10.3390/cells10030618)
Supplement: Supplementary file 1 [file cells-10-00618-s001.pdf]

cells

MDPI

Supplementary Materials:

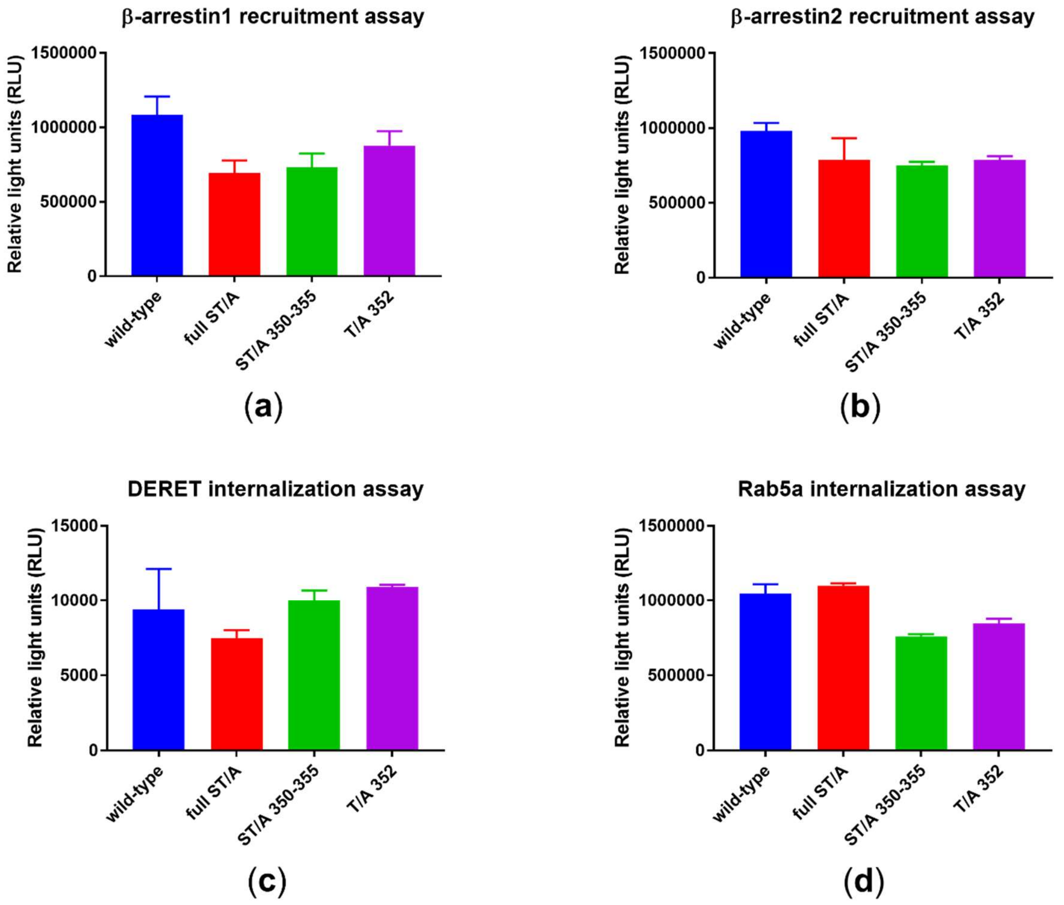

Figure 1. Expression levels of ACKR3 wild-type and mutant receptors in various assays. (**a**) Expression levels of ACKR3-RLuc wild-type and mutant receptors based on RLuc protein luminescence in a BRET β-arrestin1 recruitment assay (Figure 4a); (**b**) Expression levels of ACKR3-RLuc wild-type and mutant receptors based on RLuc protein luminescence in a BRET β-arrestin2 recruitment assay (Figure 4b); (**c**) Expression levels of SNAP-ACKR3 wild-type and mutant receptors based on Lumi4T-b fluorescence in a DERET internalization assay (Figure 5a); (**d**) Expression levels of ACKR3-RLuc wild-type and mutant receptors based on RLuc protein luminescence in a BRET Rab5a internalization assay (Figure 5b). Representative data of three independent experiments with triplicates shown as mean ± SD.
